# A national study of psychiatry outpatient visits by lower-skilled male migrant workers in Qatar

**DOI:** 10.5339/qmj.2024.29

**Published:** 2024-08-08

**Authors:** Javed Latoo, Ovais Wadoo, Yousaf Iqbal, Faisal Khan, Khizara Amin, Sami Ouanes, Shuja Reagu, Majid Alabdulla

**Affiliations:** 1 Department of Psychiatry, Hamad Medical Corporation, Doha, Qatar; 2 College of Medicine, Qatar University, Doha, Qatar; 3 Weill Cornell Medicine, Ar-Rayyan, Qatar

**Keywords:** Psychiatric morbidity, lower-skilled migrants, Arab countries

## Abstract

**Background:**

: Arab countries host 10% of all migrants globally. Migrant workers are known to have a high burden of physical and psychiatric morbidity. Most of the published literature on mental health among migrant workers is from non-Arab countries. The limited literature on migrant workers’ mental health in Arab countries is a critical research gap. It is pertinent to study well-defined migrant groups within well-defined host country conditions to yield pragmatic answers to inform service delivery.

**Aims:**

: The current study aims to complement existing data by characterizing psychiatric morbidity in a well-defined migrant group within a specifically defined context of migration.

**Methods:**

: Retrospective review of patient notes.

**Results:**

: All participants were men, and most of them were aged between 30 and 49 years. More than two-thirds presented with anxiety or depressive disorders. More than half had a past psychiatric history. Psychological distress was linked to stressors such as limited social support, living away from family, financial stressors, family-related stressors, and work-related stress. One-fourth of the participants reported stress related to the pandemic. Half of them reported physical health comorbidities. Two-thirds were not compliant with treatment plans, and one-fourth were lost to follow-up.

**Conclusion:**

: This is the first study to provide insight into the psychiatric morbidity of lower-skilled migrants presenting to outpatient psychiatry clinics in Qatar. The psychiatric morbidity of migrants is mainly centered around depressive and anxiety-related disorders. The most common challenges encountered in the management of patients include patient concordance with medication and loss to follow-up. Mitigation strategies are vital for ensuring the psychological well-being of migrant workers.

## 1. BACKGROUND

The International Labor Organization estimates that 164 million people are migrant workers; most are men (96 million), while 68 million are women. Nearly 61% of migrant workers are found in three subregions: 23.0% in North America, 23.9% in Europe, and 13.9% in Arab countries.^1^ The largest group of migrant workers in Arab countries are from South Asia (India, Pakistan, Bangladesh, Nepal, and Sri Lanka), Southeast Asia (the Philippines) and Africa (Egypt, Ethiopia, Kenya, and Uganda).^2^ Male migrant workers outnumber female migrants due to the high demand and labor opportunities in the construction sector in Arab countries. Migrant workers are known to have a high burden of physical and psychiatric morbidity. Research on the mental health of migrants in Arab countries is lacking, despite the notable population of migrants residing within this region.^3,4,5,6^ Most of the published literature on mental health among migrants is from non-Arab countries. In a systematic review of migrant workers’ psychological health, the studies included were mainly from the United States and China and reported a wide range of mental health issues, including anxiety, depression, alcohol or substance abuse, and poor sleep quality.^3^ The meta-analysis, involving 44,365 migrant workers in 17 different countries, reported an overall prevalence of depression and anxiety among migrant workers at 38.99% and 27.31%, respectively. Occupational factors, in addition to social factors, were found to be important determinants of mental health outcomes.^4^ This review included a total of 27 studies, with most studies being cross-sectional. There was a notable increase in the prevalence in comparison to a decade ago, where the reported prevalence of depression and anxiety among labor migrants was 20% and 21%, respectively.^7^ This could be influenced by the increasing trends of working-related migration in the last 10 years. This review also identified the high prevalence of stress, including acculturation stress and post-traumatic stress disorder, among migrant workers. Acculturation stress is known to be a common issue among migrant laborers.^8^ This review identified factors such as age, health issues, family history of psychiatric disorders, poor coping skills, workplace psychosocial stressors, poor working conditions, salary, abuse, limited access to healthcare, duration of residence, living conditions, and limited social support to be associated with a mental health outcome in migrant workers. It is pertinent to note that the data in this systematic review pertained to 17 different countries, but none from any Arab country. The limited literature on migrant workers’ mental health in Arab countries is mainly descriptive and contains limited quantitative information or data; nonetheless, it highlights that migrant workers are at greater risk than the native population of having psychiatric symptoms, psychiatric hospitalization, and higher suicide rates.^9,10,11^

Human migration is a complex, multidimensional phenomenon driven by and impacted by a multitude of social, cultural, economic, political, and health factors, among others.^12,13,14^ The interaction of these factors, arising from the host country, the country of origin, and the individual, determines whether the migration is a positive or negative life experience for an individual. It follows that the effect of migration on mental health is not a question that can be asked without qualifications, as the answers are often unequivocal and unhelpful.^15,16,17,18^ Therefore, framing this question within well-defined migrant groups within well-defined host country conditions is more likely to yield pragmatic solutions that can inform service delivery. Qatar is a small peninsular state that, in addition to being one of the wealthiest states, holds the unique distinction of hosting one of the highest proportions of migrants to native populations in the world. State-funded primary care centers and hospitals deliver healthcare services to both citizens and migrants in Qatar.^19,20^ Numerous initiatives and legislative changes have been implemented over time to enhance access to care.^21,22,23,24,25,26,27,28^

Along with a few of its immediate neighbors, Qatar is experiencing a rapid development phase of unprecedented growth funded by the region’s oil and natural gas reserves. This situation, in fact, does lend itself to a more specific set of conditions that can yield data by looking at a specific group of migrants who share many of the determinants known to influence both reasons for migration and mental health. These conditions are Qatar’s need for low-skilled laborers to build rapidly expanding infrastructure, a somewhat uniform background of the culture of South Asian countries that provides such labor, and uniform profiles of young men with limited skills who migrate without their families. The limited literature on migrant workers’ mental health in Arab countries is a critical research gap. The current study was mainly descriptive and exploratory and aimed to complement existing data by characterizing psychiatric morbidity in a well-defined migrant group within a specifically defined context of migration using robust clinical data. It offers a broad clinical picture of the psychiatric problems presented to psychiatry clinics. These data should assist with service planning and future research.

## 2. METHODS

### 2.1. Setting

This study was conducted at Hazm Mebaireek General Hospital (HMGH), a secondary healthcare facility run by the Hamad Medical Corporation (HMC). The HMGH is situated in the Doha Industrial Area (DIA), the capital of Qatar. The DIA is exclusively populated by lower-skilled migrant workers, with a total population of 4,25,000. Psychiatry outpatient services at HMGH, for lower-skilled male manual migrant workers, receive referrals from the Qatar Red Crescent Service, primary care services for lower-skilled migrants, the Emergency Department, inpatient units, and other outpatient services at HMGH and other hospitals associated with the HMC. Consultant psychiatrists completed all the psychiatric assessments. Patients are assessed face–to-face or virtually through a video or phone call. Psychiatrists at the HMGH are qualified Western multilingual psychiatrists who speak the same language as most patients; otherwise, translators are used.

### 2.2. Design, approval, and study population

The design was a retrospective review of electronic patient records taken in psychiatry outpatient clinics for lower-skilled migrant workers at HMGH. The study received approval from the HMC Institutional Review Board (IRB) (MRC-01-21-206). Individual patient consent was not deemed necessary by the IRB. While retrospective studies generally do not necessitate obtaining retrospective consent, securing consent for retrospective data can prove challenging, rendering it nearly unattainable. Hence, the IRB plays a pivotal role in assessing the necessity for consent. In our study, we pursued IRB approval, and the requirement for consent was waived, as the research was determined to entail minimal risk and would not adversely impact the rights and welfare of the subjects.^18^

Registers kept by the psychiatry team at HMGH were reviewed to identify the patients who met the following inclusion criteria:

1. Over the age of 18.
2. Patients who are COVID-19-negative.
3. Lower-skilled manual migrant workers referred to psychiatry outpatient clinics at HMGH between July 2019 and December 2020.
4. Patients who attended psychiatric outpatient clinics following a referral.

### 2.3. Data collection

The data collection procedure was designed to collect retrospective information from electronic medical records. Two research team members, KA and FK, extracted the data and held regular discussions to ensure consistency under the supervision of YI. The proforma collected demographic characteristics, physical comorbidity and medication details, past psychiatric history, and reasons for referral. The details of the psychiatric assessments performed by the consultant psychiatrists included the presence of psychiatric symptoms or signs, a history of substance misuse, a family history of mental illness, social stresses, a clinical diagnosis, a management plan, and any follow-up, as recorded in the notes. The diagnoses reflected clinical judgment by the psychiatrists and were not operationally defined. The data, including initial assessments and follow-up reviews, were extracted for the current outpatient journey.

### 2.4. Statistical analysis

We used Microsoft Excel 2019 for Windows to enter the data. Microsoft Excel 2019 for Windows and SPSS version 26 for Windows were used to analyze the data. We determined the absolute and relative frequencies (percentages) for each studied categorical variable.

## 3. RESULTS

Three hundred seventy-six (376) patients qualified to be part of this study over the designated study period. As expected, the population was somewhat homogenous, and most participants (73.1%, *n* = 275) were aged between 30 and 49 years. Most (77.4%, *n* = 291) were from the Indian subcontinent. The most common primary languages spoken were Hindi (56.6%, *n* = 213), English (13%, *n* = 49), and Arabic (10.6%, *n* = 40). Almost half (48.6%, *n* = 154) had physical comorbidities, and 14.2% (*n* = 40) had a positive family history. The sociodemographic and clinical features of the participants are detailed in Table 1.

Just over half (54.9%, *n* = 184) had a past psychiatric history, mostly of anxiety (54.9%, *n* = 101) or a depressive disorder (25.5%, *n* = 47). The most common sources of referral were the Qatar Red Crescent Society (47.6%, *n* = 179) and the Emergency Department (26.9%, *n* = 101). To understand the results, it is important to have a good understanding of the healthcare system in Qatar. Qatar Red Crescent Society is the primary care service provider for the lower-skilled migrants, thus explaining the common source of referrals. The most common referral symptoms were anxiety (32.4%, *n* = 122), sleep disturbances (31.4%, *n* = 118), and depression (17.8%, *n* = 67). The results of our research indicate that migrants are more likely to suffer from anxiety and depression. Fifty-nine (59, 15.7%) did not attend the first psychiatric assessment. Approximately two-thirds of the consultations were conducted through telepsychiatry. The most prevalent symptoms elicited by the psychiatrist were anxiety (48.9%, *n* = 184), sleep disturbances (36.2%, *n* = 136), low mood (18.6%, *n* = 70), and excessive worries (12.8%, *n* = 48). The most common symptoms are detailed in Table 2.

The most prevalent psychiatric disorders were anxiety disorders (38.8%, *n* = 123) and depressive disorders (28.1%, *n* = 89), and the least prevalent disorders were bipolar disorders (4.4%, *n* = 14), and substance-related disorders (4.1%, *n* = 13). The most prevalent psychiatric disorders are detailed in Table 3.

Approximately one-quarter (25.4%, *n* = 67) of the participants reported stress related to the pandemic, and most (86.4%, *n* = 274) reported social stressors. The most commonly highlighted social stressors included limited social support (22.7%, *n* = 72), living away from family (22.4%, *n* = 71), financial stressors (15.1%, *n* = 48), family-related stressors (12.0%, *n* = 38), and work-related stress (11.0%, *n* = 35).

After the initial assessment, admission was indicated for only five patients (1.6%). A pharmacological intervention was initiated in 87.7% (*n* = 278) of the patients. The most commonly prescribed drugs were escitalopram (39.1%, *n* = 124), mirtazapine (16.1%, *n* = 51), olanzapine (13.6%, *n* = 43), sertraline (8.2%, *n* = 26), and paroxetine (3.5%, *n* = 11). A non-pharmacological intervention was carried out in almost all patients (98.7%, *n* = 313). The most common non-pharmacological interventions included psychoeducation (65.5%, *n* = 205) and relaxation techniques (16.6%, *n* = 52). Most participants were offered a follow-up appointment (94.6%, *n* = 300). Approximately 91.7% (*n* = 275) of the patients attended the second appointment. The outcome was marked by complete resolution in 42.5% (*n* = 117) of patients, partial resolution (48.7%, *n* = 134), or worsening of symptoms (8.7%, *n* = 24). The most common challenges in management included patient concordance with medication (73.1%). *n* = 163) or were lost to follow-up (23.3%, *n* = 52).

## 4. DISCUSSION

To our knowledge, this is the most comprehensive review of the mental health symptoms and diagnoses of lower-skilled migrants presenting to psychiatry outpatient clinics in an Arab country. It is widely recognized that migrant workers are frequently employed in hazardous, strenuous, and precarious occupations, thereby heightening their susceptibility to workplace environmental risks. Reports indicate that migrant workers often endure substandard working conditions characterized by meager wages, extended working hours, limited job security, and instances of workplace exploitation. The compounded effects of occupational hazards and adverse work environments exacerbate the vulnerability of migrant workers to adverse health outcomes, particularly concerning their mental well-being.

More than two-thirds of the lower-skilled migrant workers in our study presented with anxiety or depressive disorders. It is well known that an increased frequency of anxiety and depression generally occurs in populations known to be under high stress, such as immigrants. A higher risk of anxiety and depression among migrant workers may be caused by elements like the experience of migration itself, exposure to stressors during the migration process, subpar living and working conditions, language barriers, restricted access to healthcare, and social isolation. A previous study indicated the difference in stressors experienced by migrant workers according to gender and working industry.^29^ The prevalence of various mental disorders in the published literature in Arab countries varies depending on the setting in which the study was conducted. In Kuwait, severe reactions to stress, manic episodes, depressive episodes, and acute and transient psychotic disorders are the most common disorders reported in an inpatient setting. Two-thirds of admitted housemaids presented with stress, anxiety, or depressive disorders. Psychiatric morbidity was 1.86 times greater in immigrant housemaids than in Kuwaiti female patients hospitalized during the 2-year study period.^30,31^ In the UAE, the prevalence of depressive symptoms among workers living in labor camps was 25.1%. Approximately 6% of participants in this cross-sectional study reported thoughts of suicide, and 2.5% had attempted suicide.^32^ In Saudi Arabia, the prevalence of depressive symptoms was found to be 20% in a community sample of low-income migrants.^33^ In Qatar, Khaled and Gray compared the prevalence of depressive symptoms among migrants and non-migrants and reported that the odds of depression significantly increased among labor migrants.^34^ In Lebanon, 66.7% of hospitalized female foreign domestic workers were diagnosed with brief psychotic episodes. Sexual, physical, and verbal abuse were reported by 12.5%, 37.5%, and 50.0% of workers, respectively.^35^ The psychiatric morbidity of migrants mostly centers around depressive and anxiety-related disorders rather than more severe and enduring mental disorders. We believe that there are several reasons for this. First, self-selection by individuals with a desire to migrate for economically productive reasons is followed by selection by companies recruiting individuals without serious mental illness. The psychological distress experienced by migrant workers leading to depressive and anxiety-related disorders is commonly linked to stressors such as an unpredictable work environment, job insecurity, living away from family and friends, language and geographical barriers, abuse, and exploitation. Similar factors, including limited social support, living away from family, financial stressors, family-related stressors, and work-related stress, were identified among the migrant workers in our study. It is pertinent to note that, as our study was performed during the COVID-19 pandemic, one-fourth of the participants reported stress related to the pandemic. Migrants were at increased risk of adverse outcomes during the pandemic.^36,37^ All our participants were men, and most of them were aged between 30 and 49 years. This finding is consistent with the overall demographic data of lower-skilled migrant workers in Qatar, who constitute more than 50% of the population. Despite the younger age group of our sample, half of them reported physical health morbidity, and 84.6% reported social stress. This finding is consistent with the notion that social stresses and physical health morbidities are common in migrants and increase the risk of mental health problems.^38^ The findings indicate that pre-existing physical illness was linked to psychological morbidity in the study population. The odds of experiencing psychological distress are greater among migrants suffering from chronic diseases such as diabetes and hypertension than among those who are not.^39,40^ More than half of our sample had a past psychiatric history, mainly of anxiety and depressive disorders. The likelihood of an individual experiencing mental health issues is greater when they have a past psychiatric disorder. The most common challenges encountered in managing patients included patient concordance with medication and loss to follow-up. Two-thirds were not compliant with treatment plans, and one-fourth were lost to follow-up. Migrant workers commonly experience financial limitations and a lack of insurance coverage, which constrains health-seeking behavior among migrant workers.^41,42^

## 5. STRENGTHS AND LIMITATIONS

This study is a first step toward highlighting the mental health needs of lower-skilled male migrant workers and will help to organize services and interventions in accordance with the type and severity of mental disorders in this patient group. Our approach was mainly descriptive and exploratory; nonetheless, the findings might be generalizable to other neighboring Arab countries with similar demographic and sociocultural contexts. The main limitation is that this was a retrospective case-note review, and the data captured were dependent on the quality of the medical records. The retrospective examination of medical records is a valuable methodological approach in clinical research; however, it is accompanied by inherent limitations. These encompass deficiencies in documentation completeness, encompassing instances of absent charts and unrecoverable or unrecorded data. Furthermore, challenges arise in the interpretation of information within the documents, hindered verification of data accuracy, and the establishment of causal relationships. Additionally, variability in the quality of information recorded by healthcare professionals poses a significant concern.^43^ No data are available for lower-skilled female migrants, as psychiatry clinics at HMGH only receive referrals for male patients. Future research should employ prospective epidemiological designs and incorporate standardized diagnostic assessments. The efforts to include diverse migrant populations, including women and higher-skilled workers, would contribute to a more comprehensive understanding of mental health issues in migrant communities.

## 6. CONCLUSION

This is the first study to provide insight into the psychiatric morbidity of lower-skilled male migrants presenting to outpatient psychiatry clinics in Qatar and other Arab countries. These findings are consistent with published evidence; however, the most important issues highlighted are non-compliance and loss to follow-up. This could be explained by financial reasons, the stigma of taking psychiatric medications, a lack of personal transport to attend appointments, the inability to seek permission from the employer for hospital appointments, and cultural beliefs about mental illness.

Efforts to ameliorate the mental health challenges faced by lower-skilled migrant workers necessitate a multifaceted approach. Primarily, there is a critical need for comprehensive workplace reforms aimed at enhancing working conditions, such as implementing fair wages, reasonable working hours, and stringent measures to prevent workplace abuse. Additionally, proactive measures to mitigate occupational hazards through robust health and safety protocols are imperative. Introducing support mechanisms within workplaces, such as counseling services and mental health awareness programs, can provide crucial assistance to those struggling with psychological distress. Moreover, policies facilitating access to healthcare services and social support networks are pivotal in bolstering the resilience of migrant workers. Collaborative efforts between governmental agencies, employers, civil society organizations, and migrant communities are essential for fostering an environment that prioritizes the mental well-being of lower-skilled migrants. By addressing systemic challenges and promoting a culture of inclusivity and support, strides can be made towards fostering healthier and more equitable working environments for all migrant workers.

## CONFLICT OF INTEREST STATEMENT

All authors declare that the research was conducted in the absence of any commercial or financial relationships that could be construed as potential conflicts of interest.

## AUTHORS’ CONTRIBUTIONS

JL, OW, YI, and MA conceived the idea for the research. KA and FK collected the data under the supervision of YI. SO, SR, and OW analyzed the data. JL and OW wrote the initial draft of the article. The other authors critically appraised the article and provided input into the final manuscript. All the authors approved the final manuscript.

## DATA AVAILABILITY STATEMENT

The authors have made all the information needed to draw the conclusions in this research accessible. Any legitimate request will result in access to all the data from the corresponding author.

## ETHICS APPROVAL AND CONSENT TO PARTICIPATE

The study received approval from the HMC Institutional Review Board (IRB) (MRC-01-21-206). Individual patient consent was not deemed necessary by the IRB.

## Figures and Tables

**Table 1:** Sociodemographic and clinical features of the participants.

		*n*	%
Age range	<20	1	0.3%
	20–29	66	17.6%
	30–39	171	45.5%
	40–49	104	27.7%
	50–59	22	5.9%
	60–69	9	2.4%
	70–79	2	0.5%
	80–89	1	0.3%
Ethnicity	Indian	112	29.8%
	Pakistani	30	8.0%
	Bengali	73	19.4%
	Nepali	62	16.5%
	Srilankan	14	3.7%
	Filipino	5	1.3%
	African	15	4.0%
	Qatari	4	1.1%
	Other Arabs	51	13.6%
	Other	10	2.7%
Primary language spoken	English	49	13.0%
	Hindi	213	56.6%
	Urdu	24	6.4%
	Bengali	5	1.3%
	Nepali	1	0.3%
	Telugu	1	0.3%
	Pashto	4	1.1%
	Arabic	40	10.6%
	Others	8	2.1%
	Unknown	31	8.2%
Any physical comorbidities		154	48.6%
Recent history of substance misuse		35	10.7%
Past psychiatric history		184	54.9%
Family history		40	14.2%

**Table 2.** Prevalence of psychiatric signs and symptoms reported (most common).

		*n*	%
Referral symptoms	Anxiety	122	32.4%
	Sleep disturbances	118	31.4%
	Depression	67	17.8%
	Somatic symptoms	39	10.4%
	Others/stress/facial tics	33	8.8%
	Paranoid thoughts	29	7.7%
	Palpitations	22	5.9%
	Excessive worries	16	4.3%
	Negative thoughts	15	4.0%
	Low energy/tiredness/fatigue	12	3.2%
	Irritability	12	3.2%
Symptoms elicited by thepsychiatrist	Anxiety	184	48.9%
	Sleep disturbances	136	36.2%
	Low mood	70	18.6%
	Excessive worries	48	12.8%
	Dysphoria/anhedonia	38	10.1%
	Panic-like symptoms	38	10.1%
	Reduced appetite	31	8.2%
	Irritability	25	6.6%

**Table 3.** Most prevalent psychiatric diagnosis.

		*n*	%
Type of diagnosis	Anxiety disorders	123	38.8%
	Depressive disorders	89	28.1%
	Somatic symptom and related disorders	19	6.0%
	Schizophrenia spectrum and other psychotic disorders	17	5.4%
	Trauma and stress-related disorders	15	4.7%
	Bipolar and related disorders	14	4.4%
	Substance-related and addictive disorders	13	4.1%
	Sleep and wake disorders	12	3.8%
	Obsessive-compulsive and related disorders	8	2.5%
	Neurocognitive disorders	5	1.6%
	Personality disorder	2	6.3%
	No mental illness	16	5.0%
